# The Performance of mHealth in Cancer Supportive Care: A Research Agenda

**DOI:** 10.2196/jmir.3764

**Published:** 2015-02-13

**Authors:** Greta Nasi, Maria Cucciniello, Claudia Guerrazzi

**Affiliations:** ^1^Department of Policy Analysis and Public ManagementBocconi UniversityMilanoItaly; ^2^Public Management and Policy DepartmentSDA Bocconi School of ManagementMilanoItaly; ^3^Center for Research in Health and Social Care Management (CeRGAS)Bocconi UniversityMilanItaly; ^4^Department of Health Services AdministrationSchool of Health ProfessionsUniversity of Alabama at BirminghamBirmingham, ALUnited States

**Keywords:** mHealth, performance, organizational performance, efficiency, effectiveness, clinical effectiveness, quality of life

## Abstract

**Background:**

Since the advent of smartphones, mHealth has risen to the attention of the health care system as something that could radically change the way health care has been viewed, managed, and delivered to date. This is particularly relevant for cancer, as one of the leading causes of death worldwide, and for cancer supportive care, since patients and caregivers have key roles in managing side effects. Given adequate knowledge, they are able to expect appropriate assessments and interventions. In this scenario, mHealth has great potential for linking patients, caregivers, and health care professionals; for enabling early detection and intervention; for lowering costs; and achieving better quality of life. Given its great potential, it is important to evaluate the performance of mHealth. This can be considered from several perspectives, of which organizational performance is particularly relevant, since mHealth may increase the productivity of health care providers and as a result even the productivity of health care systems.

**Objective:**

This paper aims to review studies on the evaluation of the performance of mHealth, with particular focus on cancer care and cancer supportive care processes, concentrating on its contribution to organizational performance, as well as identifying some indications for a further research agenda.

**Methods:**

We carried out a review of literature, aimed at identifying studies related to the performance of mHealth in general or focusing on cancer care and cancer supportive care.

**Results:**

Our analysis revealed that studies are almost always based on a single dimension of performance. Any evaluations of the performance of mHealth are based on very different methods and measures, with a prevailing focus on issues linked to efficiency. This fails to consider the real contribution that mHealth can offer for improving the performance of health care providers, health care systems, and the quality of life in general.

**Conclusions:**

Further research should start by stating and explaining what is meant by the evaluation of mHealth’s performance and then conduct more in-depth analysis in order to create shared frameworks to specifically identify the different dimensions of mHealth’s performance.

## Introduction

Health care is undergoing an evolutionary phase worldwide aimed at facing multiple challenges: (1) the aging global population is increasingly affected by chronic diseases for much longer [1,2], (2) health care delivery costs are becoming unsustainable [3], (3) societies are becoming more and more mobile [4], and (4) being cared for at home is increasingly the preferred mode of health care delivery [2,5]. The unsustainability of current health care spending has led to the need for disruptive solutions, capable of controlling costs without diminishing quality of service and quality of life.

Chronic diseases are becoming the heaviest burden on health care systems worldwide, and cancer is one of these. A chronic disease can be defined as a condition that lasts a year or longer and requires ongoing monitoring and treatment [6]. Although cancer continues to be one of the main causes of death, efforts have been made in several fields of medicine in order to reduce cancer mortality every year [7].

This scenario has witnessed the rapid and ongoing growth in mobile technologies, especially mobile health (mHealth) defined as “medical and public health practice supported by mobile devices, such as mobile phones, patient monitoring devices, personal digital assistants (PDAs), and other wireless devices” [8]. According to this definition, mHealth includes short messaging services (SMS) as well as more complex applications like general packet radio service (GPRS), third and fourth generation mobile telecommunications (3G and 4G systems), global positioning systems (GPS), and Bluetooth technology [8].

Furthermore, major advances have been carried out into two subfields: wearable and body area sensor networks, and mobile broadband and wireless Internet mHealth systems [9]. An example of the former is the innovative WE-CARE system: an intelligent telecardiology system that exploits mobile wireless networks in order to provide benefits in detection rate and time savings [10]. An example of the second subfield is the concept of 4G health. The introduction of the fourth-generation mobile communication system led to a turning point and “the evolution of mHealth towards targeted personalized medical systems with adaptable functionalities and compatibility with the future 4G networks” [9]. The prospect of managing health care via mobile platforms has resulted in a momentous technology drive and the implementation of thousands of mobile apps, mainly designed for a single condition or aspect of disease management.

Over the past decade, especially since the advent of smartphones, mHealth has come to the attention of the health care system as something that could radically change the way health care has been viewed, managed, and delivered to date. By exploiting their technical capabilities, mobile phones can be used to implement several health care interventions, ranging from increasing the accessibility of health care information (eg, short messages or reminders) to involving the health care team (eg, remote monitoring) [11]. To this extent, a mobile phone with a wireless connection is an essential prerequisite because, as Huang et al state: “a wireless network may be not mobile, but a mobile network must be wireless” [10]. In the case of cancer, hundreds of apps have already been designed and implemented with several purposes, such as raising awareness about chronic disease, providing information about cancer, or for managing cancer [12].

mHealth has generated a surge of positivistic policy documents, such as the Digital Agenda for Europe [13] and the Federal Health IT Strategic Plan [14] in the United States, and this emerging industry has attracted large investments. mHealth makes it possible to follow the shifting focus of health care from “cure” to “care” thanks to its tendency to support the entire care process, including wellness and prevention. This is important in the case of cancer, one of the leading causes of death worldwide, accounting for 8.2 million deaths in 2012 [15].

mHealth may play a particularly significant role in cancer supportive care, dealing with the management of the side effects of cancer treatment, since patients and caregivers play a role in managing side effects and, given adequate knowledge, are able to demand appropriate assessments and intervention. In this scenario, mHealth has great potential for linking patients, caregivers, and health care professionals, for enabling early detection and intervention, for cutting costs, and achieving better quality of life.

Given its huge potential, it is important to evaluate the performance of mHealth. Literature has shown that the performance of mHealth can be assessed from several perspectives. It can be seen as a return on integrated care processes, since it can improve communication and enhance integration among those involved in health care processes [16,17]. In terms of organizational performance, mHealth can increase the productivity of health care providers and possibly even the productivity of health care systems as a result [18-20]. For external relations, mHealth can enhance transparency, increasing the accountability of health care providers and systems [21,22], and it can also empower patients [1,23-25]. Finally, the greatest promise of mHealth is that it may boost the appropriateness of care and possibly the quality of life [26,27].

This paper intends to review studies on the evaluation of the performance of mHealth, with a particular focus on cancer care and cancer supportive care processes, concentrating on its contribution to organizational performance. It also aims to identify elements for a further research agenda.

## Methods

We carried out a review of papers from three bodies of literature: medical informatics, health care management, and medicine, with particular reference to oncology journals. The first step of our research strategy (Table 1) was aimed at identifying and collecting all existing studies on the evaluation of mHealth’s performance in cancer and cancer supportive care. We started by identifying a number of keywords and entered them in our selected computerized bibliographical databases, resulting in a total of 1698 papers, including 106 that were relevant in terms of mHealth and performance and were used for our assessment. We then narrowed down this result to cancer supportive care, leading to a total of 67 papers, including 15 that were useful for our analysis.

We subsequently used a “bibliographic network approach” to track the articles in the references in the works we considered fundamental for our research. We retrieved papers and studies published after 1999 in academic reviews and journals that were not listed in the database at the time of the analysis, but which were known among academics.

**Table 1:** Research strategy to identify and collect relevant studies.

Search strategy	Detailed information
Keywords	Generic search using concept words: “mHealth”, “cancer”, “quality of life”, Specific searches : “mHealth” (mHealth OR mHealth OR “mobile health” OR “mobile health care”) + “cancer” (cancer OR “cancer care” OR “cancer supportive care” OR “supportive care in cancer” OR “chemotherapy” OR “side effects” OR “adverse effects” OR “integrated care” OR “cancer integrated care”) + “Quality of life” (“quality of life” OR “quality of service” OR “quality of care” OR “health care delivery” OR “health care management” OR “care management” OR “health policy” OR promises OR “continuity of care” OR “lean health care” OR “lean health care” OR “lean thinking” OR “patient-centered”) + “performance” (“performance” OR “evaluation” OR “impact” OR “assessment” OR “return” OR “promises” OR “adoption”)
Databases	BioMed Central, Business Source Complete, IEEE Xplore, PLOS (One, Medicine and Clinical Trials), PubMed, Science Direct, Web of Science (which embeds Elsevier, Wiley, JMIR, JAMIA), Cochrane Library
Specific journals	JAMIA, JMIR, BMJ, Health affairs, Health care management review, Health Policy, Health Policy and Technology, Value in Health (ISPOR), Journal of Cancer Policy, Academy of Management Journal, Journal of Management studies, Journal of Health Economics, Health economics, Canadian Medical Association Journal, Health Informatics Journal, Journal of Clinical Oncology (ASCO), Annals of Oncology (ESMO), Supportive Care in Cancer (MASCC), European Journal of Cancer (published by Elsevier, official journal of EORTC, ECCO, EACR and EUSOMA), Critical Reviews in Oncology and Hematology (ESO), Health Services Management Review (EHMA), IEEE Journal of Biomedical and Health Informatics, IEEE Transactions on Information Technology in Biomedicine, Journal of Biomedical Informatics
Inclusion criteria	Peer-reviewed published articles
	Published since 1999
Exclusion criteria	Grey literature (blogs, newsletters, videos)
	Provisional or structured abstracts
	Poster sessions, presentations, comments, opinions, discussions, editorials, prefaces, summaries, interviews, correspondence, tutorials
	Studies focused only on (1) design of the device or the app, (2) technology (communication and Web protocols, standards, platforms), and (3) characteristics of the technology (eg, wireless technology, bandwidth, battery life, connectivity, signal quality)
	Studies on psychology, ie, behavioral models and theory of psychology
	Studies on definition of new quality of life measurements as influenced by the technology
	Studies set in low resource settings or developing countries, except when talking about implementation of new technologies in low resource settings (sustainability, etc)
	Studies where mobile health means mobile clinics or mobility of professionals or mobile screening units
	Studies or articles with no author
	Studies or articles with no abstract

## Results

### Performance of mHealth

#### Study Characteristics

Our first finding is that there is a very limited amount of literature on mHealth’s performance. Our analysis revealed that only 35.8% (38/106) of our selected papers looked at mHealth’s performance in some way, as most of the studies focused on the use of mHealth, and less on adoption and its determinants and barriers.

**Figure 1:**
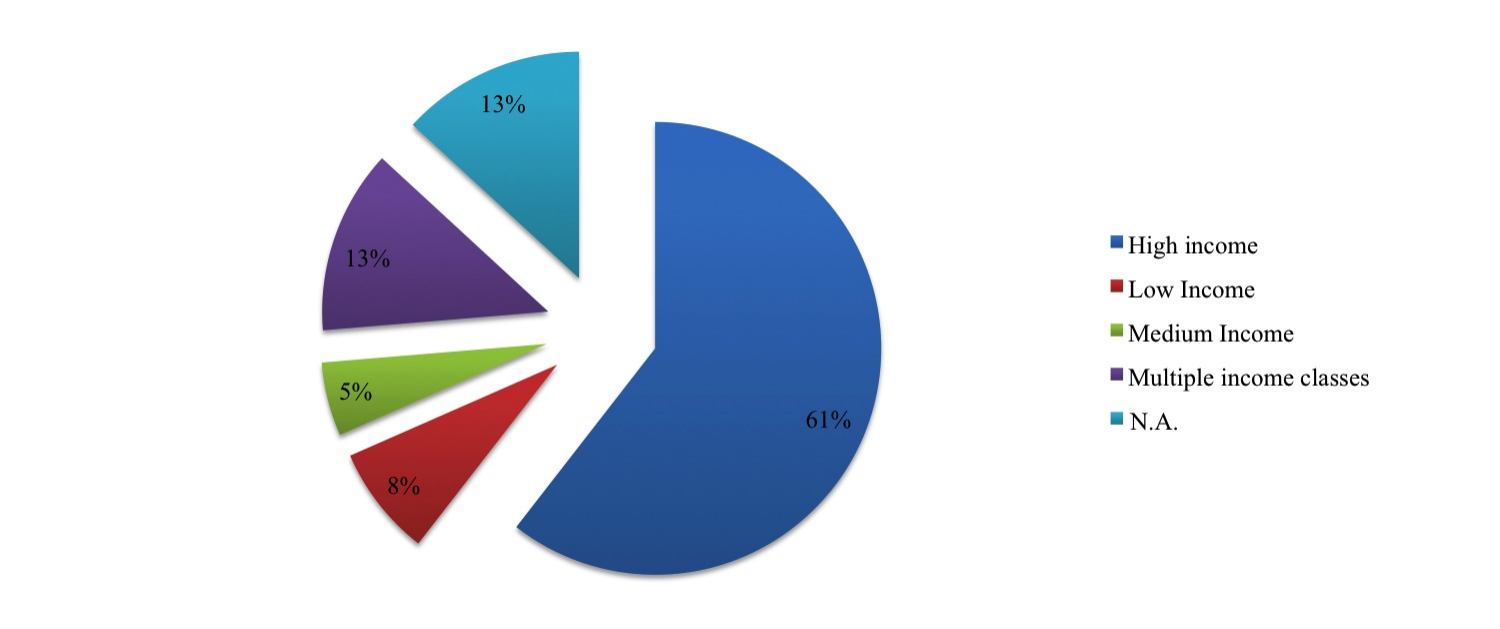
Type of country.

More frequently, studies offered an assessment of the performance of technology rather than an evaluation of the contribution to organizational performance and the quality of life of patients. According to the categorization provided by the World Bank [8], countries can be classified according to three “income-classes”: high, medium, and low income. Most studies referred to high income countries (61%, 28/38 papers) and less to low income (8%, 3/38 papers) and middle income countries (5%, 2/38 papers). We should mention that 13% of papers referred to different type of countries (5/38 papers) and 13% (5/38 papers) of our selected studies did not refer to any specific country or region (Figure 1) since their contribution was based on a literature review with no specific reference to any country.

**Figure 2:**
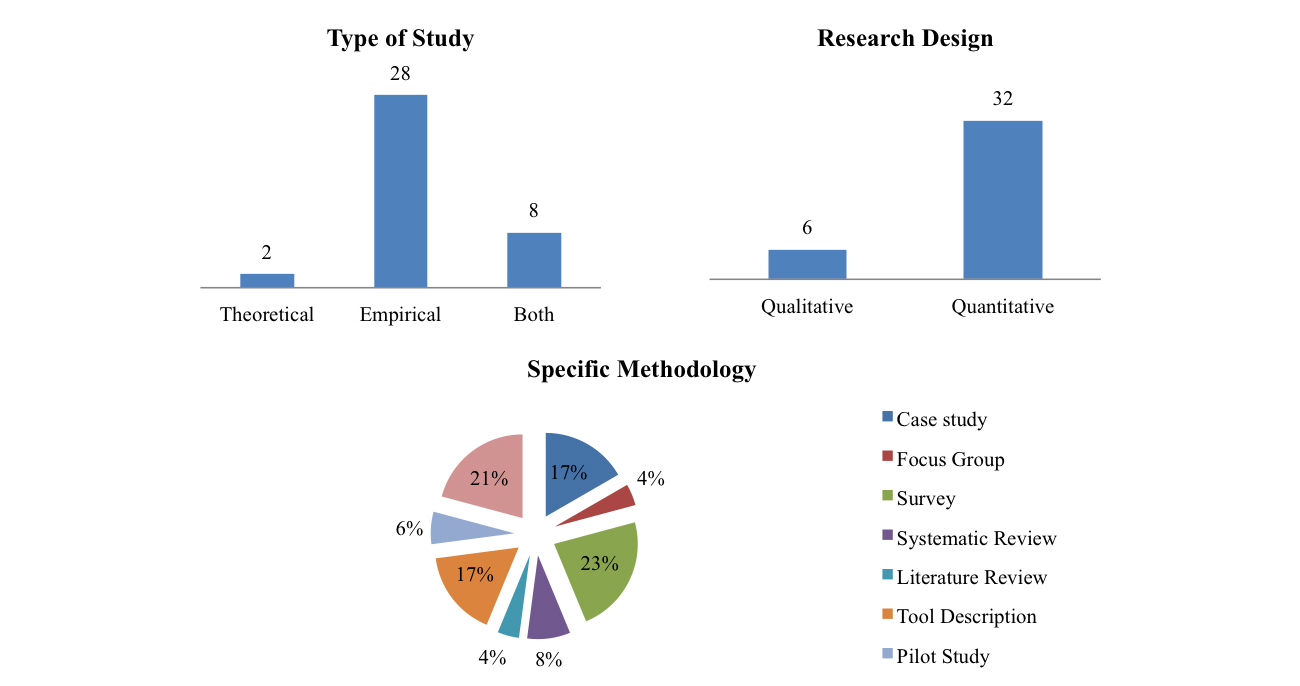
Methodology of studies.

Most papers were empirical in methodology (about 73%, 28/38 papers) (see Figure 2). Other specific methodologies were used, some taking a more qualitative research design, such as literature review, case studies, tool description, and focus groups. However, most were based on more quantitative designs, like randomized controlled trials, systematic reviews, surveys, and pilot studies.

**Figure 3:**
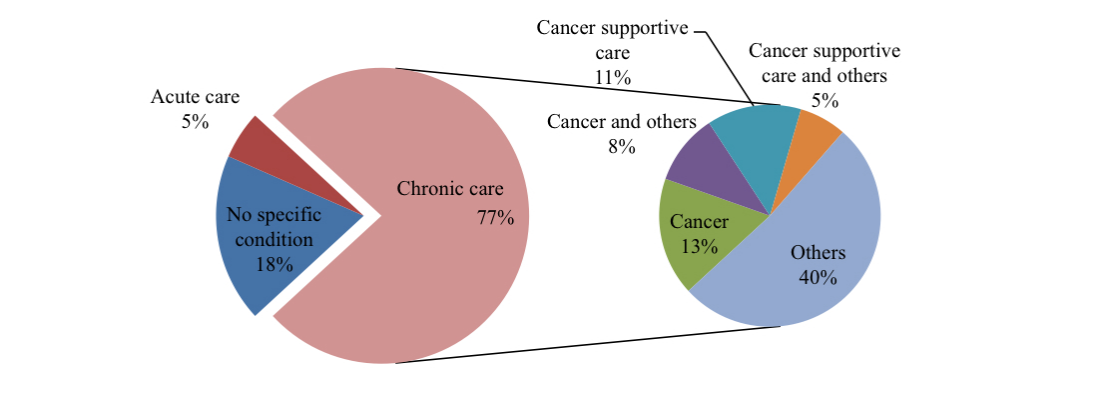
Health condition.

Looking in greater depth at the health condition under analysis (Figure 3), only 5% (2/38 papers) of the papers focused on acute care, 18% (7/38 papers) did not focus on any specific condition, and the majority of papers (77%, 29/38 papers) looked at chronic care. Chronic care includes cancer and cancer supportive care, which accounted for 37% (14/38 papers) of the papers on performance in our analysis, as well as several other diseases, such as asthma, diabetes, and obesity.

**Figure 4:**
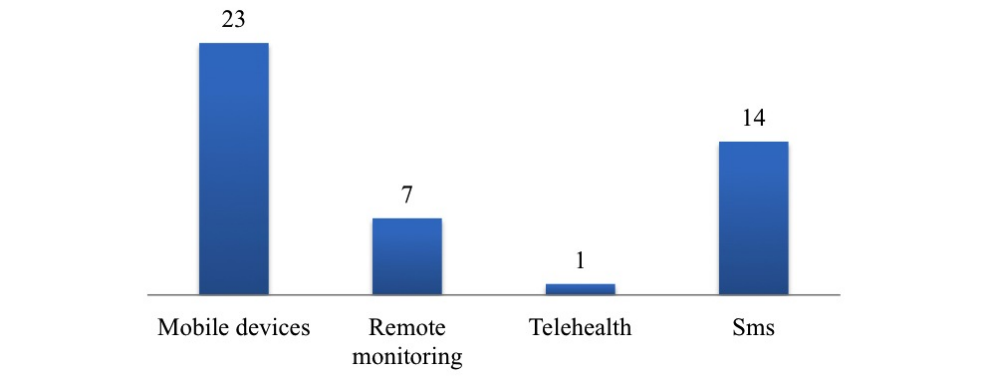
Type of technology.

Looking at the type of mobile technologies analyzed relating to performance, 61% (23/38 papers) of papers discussed mobile devices (like smartphones and tablets) and apps, 18% (7/38 papers) remote monitoring technologies, 37% (14/38 papers) SMS technologies, and only 3% (1/38 paper) focused on telehealth (Figure 4). We should mention that some papers referred to several types of mobile technologies.

#### Evaluation of mHealth’s Performance

The assessment of mHealth’s performance is based on the use of multiple measures. It is mainly measured in terms of better quality information [3,16,17,28-30]. Baumgart analyzed information-sharing, showing that the quality of information increased for certain activities as a result of using PDAs and tablets, such as billing, prescription writing, medical calculation, scheduling, and drug reference [31]. A study by Hamou et al showed that using mobile technologies for collecting patient data and feedback could promote better information when used in a clinical setting [20].

Another measure of performance often analyzed is cost savings [25,32,33]. A report by Boston Consulting Group and Telenor, for example, analyzed the role of mHealth in homecare for the elderly [34]. According to this report, costs savings for caring for the elderly varied in three different countries, with amounts ranging from €1.25 billion in Denmark to €2.4 billion in Sweden.

Other studies have shown that mHealth may make patient assessment more straightforward and less time consuming [17,35,36]. Some studies examined mHealth’s performance with respect to improvements in medical treatment adherence rates [37,38] and consequent re-hospitalization rates [39-41]. Other papers studied the quality of service [42-44], but they often had a specific and narrow scope of mHealth use (eg, mHealth offers patients improved mobility and comfort thanks to wireless technology) [45]. Some papers analyzed mHealth’s performance by looking at the enhanced monitoring of patients that also led to better disease management [46].

Using mobile technologies to collect up-to-date data can help patients regain functional independence and help hospitals determine the appropriate length of stay for a patient [47] and thus help cut the cost of hospitalization [25]. Finally, some studies have shown that health care communities created via mHealth can enhance quality of life by providing peer support, whereby patients are able to exchange opinions with regard to a certain drug, physician, therapy, or share personal experiences [27,48].

#### Discussion of Findings

Although there is not necessarily a common assessment of the measures of performance to be found in literature, they can be grouped into main dimensions based on other studies assessing innovation and technological innovations [49,50]. For instance, papers that measured performance in terms of the quality of information, cost savings, and time savings actually focused on efficiency measures [21]. Other studies related to effectiveness [39,51], and some of these focused on dimensions of organizational performance from the health care provider’s viewpoint. In particular, the focus was on cutting re-hospitalization rates or other indirect effects associated with improved integration of health care. With regard to effectiveness, another limited body of research focused on quality of service. Finally, very few papers analyzed clinical effectiveness in public health [26] and the role of mHealth for enhancing quality of life [26].

In particular, research considering quality of life focused on a single dimension, that is, enhancing the social relationships of patients. However, quality of life was typically associated with other dimensions, like a person’s physical health, psychological state, level of independence, personal beliefs, and local environment [52].

#### Performance of mHealth in Cancer Care

**Figure 5:**
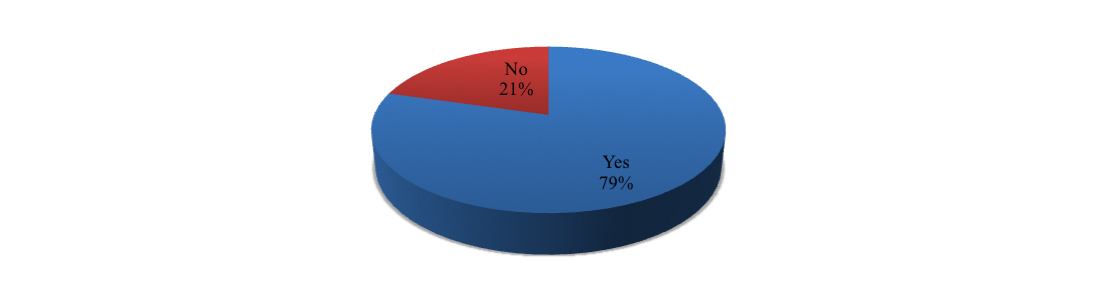
mHealth performance in cancer and cancer supportive care: empirical studies.

We subsequently studied papers assessing the performance of mHealth in cancer supportive care. Unfortunately, there were very few papers on this important care process (5.6% of total papers, 6 papers), and so we extended our analysis to include all papers assessing mHealth’s performance in cancer care. These studies were mainly empirical, and most of them identified similar measures of performance (Figure 5).

First, the dimensions discussed above must be defined [53]. The framework consists of four performance dimensions: efficiency and effectiveness, which are output measures, and clinical effectiveness and quality of life, which are outcome measures.

Efficiency focuses on the evaluation of mHealth in terms of quality of information, time saving, and cost savings. Effectiveness is related to the contribution that mHealth gives to the process of integration and improvement of patient care processes, evaluated from the health care provider’s perspective. To this extent, this dimension can be divided into two measures, namely organizational performance and quality of service.

Clinical effectiveness deals with the evaluation of the effects produced on clinical activities, such as improvements in the adherence rate to medical treatment. Finally, quality of life measurements refer to the evaluation of mHealth in terms of physical and psychological state.

**Figure 6:**
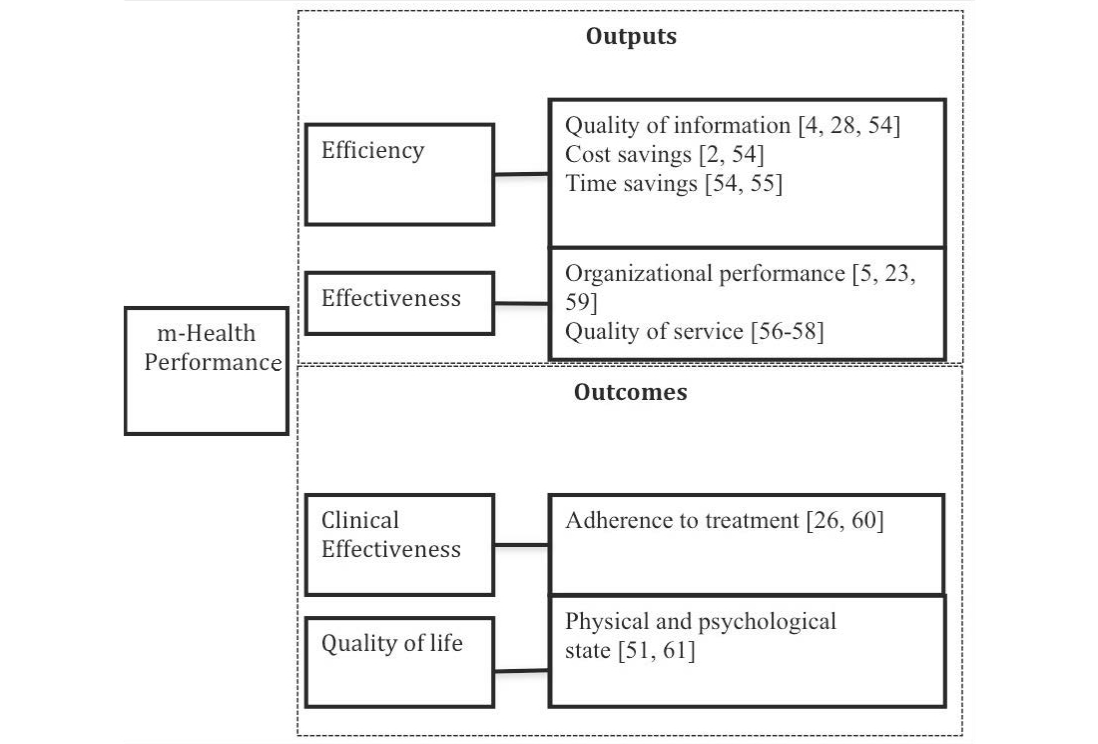
Dimensions and measures of mHealth performance in cancer care.

Figure 6 shows the different measures of performance of mHealth in cancer care grouped by these dimensions.

##### Efficiency

Most papers focused on better quality information with respect to efficiency [4,54]. A 2001 study, for example, found that many clinical procedures relating to patient management are repetitive and Workflow Management Systems for oncology can automate these repeated activities by using mobile applications to transfer data services, like remote monitoring. Workflow implies the automation of business processes in order to promote the transition of information within the organization and can enable health care institutions to transform large amounts of medical data into contextually relevant clinical information [28].

Literature on mHealth’s performance in cancer analyzed other measures of efficiency, including cost savings [2,54] and time savings [54,55]. Holzinger analyzed the impacts of a new method for collecting skin cancer data [54]. Patients filled out a questionnaire on a tablet personal computer, and the medical data collected became part of the electronic patient record made directly available to physicians. The author found this instrument generated annual savings worth up to €40,000 compared to the estimated annual cost of €55,000 if mHealth were not used.

Holzinger’s research [54] also showed another measure of efficiency, that is, time savings. His study actually found data indicating up to 90% reduction in the time needed for data entry. This may be particularly significant if mHealth can make it possible to save time by sharing information generated by central hospitals located in big cities with remote care centers [55]. Furthermore, ready access to patient data by means of mobile devices and technologies can lead to a significant reduction in medical errors.

##### Effectiveness

Quality of service is one measure of effectiveness [56-58]. Lamber et al showed how mobile technologies can help monitor oncological patients during day hospital therapies if a mobile service is an integral part of the hospital’s information system. This instrument guides patients at hospital by means of a “patient guidance service” telling them what they have to do next [56].

Many papers, however, focus on better monitoring, especially those related to cancer supportive care [5,23,59]. For example, Kearney evaluated the impact of a remote monitoring system based on mobile technology assessing the effects of six common side effects of chemotherapy [23]. In the same way, Mooney tested the feasibility of a telephone-based computerized system used for monitoring chemotherapy symptoms by generating alerts to health care providers [59]. More specifically, Mooney illustrated the usefulness of mHealth for assessing less common symptoms that are usually poorly controlled.

##### Clinical Effectiveness

Clinical effectiveness seems to be mainly related to the appropriateness of care, often measured through the adherence rate to medical treatment [26,60]. Heinrich evaluated the use of handheld devices that provide electronic reminders for medication to a sample of adults suffering chronic illness [26]. Moreover, adherence to medical treatment may be improved through a software app developed for a mobile phone platform to support regular and correct drug intake, also leading to better disease management [60].

##### Quality of Life

Finally, quality of life is a poorly investigated dimension of performance with specific reference to cancer. We found only two papers assessing this dimension, which examined several chronic conditions, including cancer [51,61]. They discussed the contribution of mHealth to a patient’s health and behavior in general terms.

## Discussion

### Principal Findings

This paper helps investigate the performance of mHealth, with particular reference to mHealth in cancer supportive care. Although there is abundant literature on mHealth, it is lacking with regard to mHealth’s performance, especially in relation to cancer and cancer supportive care. Most mHealth studies focus more on the mobile technology itself, rather than on its adoption and performance, as confirmed by Van Heerden et al [62]. However, introducing systems like mHealth for managing health care-related information is not limited to technology, since it demands the capacity to integrate technology, people, and processes.

Most papers that we reviewed focused on the use of mHealth, some looked at the performance of mHealth, and very few papers looked at the determinants of mHealth. The papers on the early stages of the innovation process actually focused on pilot projects rarely leading to wide-scale adoption. Pilot studies have been carried out, and mobile apps have been developed and tested on specific contexts. According to Tomlinson et al [63], there are more than 500 mHealth studies on pilot projects, but almost nothing is known about the likely uptake of these initiatives after the pilot projects are completed. As a result, it is clear that there are huge scaling-up problems.

If we have to determine whether mHealth can actually meet its promise, our analysis found very limited evidence when it comes to mHealth in general and its contribution to better quality of life. Research is almost always based on single studies and on a single dimension of return. For example, papers often do not analyze efficiency, but focus on cost- or time savings. Any evaluation of the performance of mHealth is based on very different methods and measures, with a prevailing focus on the quality of information. This fails to consider the contribution that mHealth may offer to improving the performance of health care providers, health care systems, and to quality of life in general.

Technically, there is little evidence of evaluation processes based on structured, solid, consistent, and mature methodologies. Furthermore, the evaluations were not part of larger and more extensive performance measurement processes, starting with defining goals for mHealth supporting a given health care process, cancer care processes in particular, and then systematically and continuously analyzing what happens next. This approach to evaluation is crucial because effects may not necessarily be evident immediately after the introduction of innovation; evaluation should be monitored over time to allow for effects that become visible in the short, medium, and long term.

The existing literature also usually involved a single specific stakeholder, whereas our vision takes in multiple types of stakeholders [64,65] working in this specific field, who all perceive benefits resulting from their involvement. Thus, all these benefits should be analyzed.

### Possible Future Research Agenda

#### Overview

This paper is a preliminary study that analyzes the performance of mHealth in cancer supportive care. To date, there is limited published research in this field. Consequently, we identified some areas for further research.

#### Systematic Review of Definitions for the Evaluation of mHealth’s Performance

A first substantial contribution to assessing mHealth’s performance would be a systematic assessment and review of the current definitions of the scope and boundaries of the evaluation processes. In the private sector, a substantial body of empirical and theoretically informed research has led to discussion on return of investment measures and key performance and success indicators. A main motivation for evaluation is the need to monitor profitability results, in turn providing an incentive for further innovation in order to cut costs and improve market share, which fits the purpose of some higher-income countries with private health care systems. However, the adoption of mHealth is generally supported for more general purposes, such as improving the efficiency, productivity, and adequacy of care services. This leads to benefits going beyond organizational results, with more social-related outputs and outcomes, and impacts such as quality of life.

#### Development of Solid Frameworks to Measure the Performance of mHealth

There is a prevailing focus on empirical studies, each adopting its own measures of performance. Theoretical studies should be carried out in order to better understand the performance of mHealth. There is a need to develop and consolidate more systematic frameworks since most studies focus on single measures of performance, although we grouped them into dimensions, with the aim of providing measurement systems of mHealth performance. This is fundamental for depicting results and for creating opportunities for comparing evidence and generalizing findings.

#### Multi-Stakeholder Expectations and Multi-Stakeholder Assessment of mHealth Performance

mHealth is a technological innovation that could affect multiple stakeholders. This has at least two implications for a research agenda. First, it suggests an investigation of the expectations of stakeholders with the purpose of prioritizing mHealth adoption where there is a need for it. This may lead to favorable and supportive opinions relating to the adoption process and to the identification of mechanisms to generate value and stimulate commitment towards mHealth. Second, evidence regarding the expectations of stakeholders should be considered in order to set goals and define targets for results that should be reflected in the measurement frameworks.

#### Systematic Performance Measurement Cycles

Most studies analyzed were based on empirical work on pilot projects and tests of mHealth adoption. Evidence [63] suggests that this does not always lead to wide-scale adoption. Literature on performance measurement [49,50] suggests that evaluating innovation performance should be an ongoing activity. Not all effects of mHealth embedded in a care process may be measurable at the same time. Most technological innovations produce multiple effects at different times after adoption [66-68], so it seems relevant to systematically and repeatedly collect data on the impacts of this innovation.

#### Methods

Some studies [43,59] are based on perceptions and limited interviews. Further research should address the question of methods that are more fit for purpose.

Finally, it seems appropriate to link the evaluation of mHealth’s performance to the scope and use of mHealth. This might provide an honest assessment of the actual contribution that mHealth can offer.

### Conclusions

Our analysis revealed that studies evaluating the performance of mHealth are based on very different methods and measures, with a prevailing focus on issues linked to efficiency. This fails to consider the real contribution that mHealth can offer for improving the performance of health care providers, health care systems, and the quality of life for patients.

## Abbreviations

mHealth: mobile health
PDA: personal digital assistant
SMS: short message service
